# Estimation of screening test (Hemoccult®) sensitivity in colorectal cancer mass screening

**DOI:** 10.1054/bjoc.2001.1752

**Published:** 2001-06

**Authors:** J L Jouve, L Remontet, V Dancourt, C Lejeune, A M Benhamiche, J Faivre, J Esteve

**Affiliations:** Burgundy Digestive Tract Cancer Registry (INSERM CRI 9605), Faculty of Medicine, 7 bd Jeanne-d'Arc, BP 87900, 21079 Dijon cedex, France; Biostatistical Unit, CHU Lyon-Sud, 69494 Pierre-Bénite cedex, France

**Keywords:** colorectal neoplasm, mass-screening, sensitivity, sojourn time, statistical models

## Abstract

3 controlled cohorts of mass-screening for colorectal cancer using a biennial faecal occult blood (HemoccultII®) test on well-defined European populations have demonstrated a 14% to 18% reduction in specific mortality. We aimed to estimate the sensitivity (S) of this HemoccultII®test and and also mean sojourn time (MST) from French colorectal mass-screening programme data. 6 biennial screening rounds were performed from 1988 to 1998 in 45 603 individuals aged 45–74 years in Saône-et-Loire (Burgundy, France). The prevalent/incidence ratio was calculated in order to obtain a direct estimate of the product S.MST. The analysis of the proportional incidence and its modelling was used to derive an indirect estimate of S and MST. The product S.MST was higher for males than females and higher for left colon than either the right colon or rectum. The analysis of the proportional incidence confirmed the result for subsites but no other significant differences were found. The sensitivity was estimated at 0.57 and the MST at 2.56 years. This study confirms that the sensitivity of the Hemoccult test is relatively low and that the relatively short sojourn time is in favour of annual screening. © 2001 Cancer Research Campaign http://www.bjcancer.com


British Journal of Cancer (2001) 84(11), 1477–1481
© 2001 Cancer Research Campaign
doi: 10.1054/ bjoc.2001.1752, available online at http://www.idealibrary.com on IDEAL®

http://www.bjcancer.com

### Estimation of screening test (Hemoccult®) sensitivity in colorectal cancer mass screening

**JL Jouve[1], L Remontet[2], V Dancourt[1], C Lejeune[1], AM Benhamiche[1], J Faivre[1] and J Esteve[2]**

[1]Burgundy Digestive Tract Cancer Registry (INSERM CRI 9605), Faculty of Medicine, 7 bd Jeanne-d'Arc, BP 87900, 21079 Dijon cedex, France; [2]Biostatistical Unit, CHU Lyon-Sud, 69494 Pierre-Bénite cedex, France

**Summary** 3 controlled cohorts of mass-screening for colorectal cancer using a biennial faecal occult blood (HemoccultII®) test on well-defined European populations have demonstrated a 14% to 18% reduction in specific mortality. We aimed to estimate the sensitivity (S) of this HemoccultII® test and and also mean sojourn time (MST) from French colorectal mass-screening programme data. 6 biennial screening rounds were performed from 1988 to 1998 in 45 603 individuals aged 45–74 years in Saône-et-Loire (Burgundy, France). The prevalent/incidence ratio was calculated in order to obtain a direct estimate of the product S.MST. The analysis of the proportional incidence and its modelling was used to derive an indirect estimate of S and MST. The product S.MST was higher for males than females and higher for left colon than either the right colon or rectum. The analysis of the proportional incidence confirmed the result for subsites but no other significant differences were found. The sensitivity was estimated at 0.57 and the MST at 2.56 years. This study confirms that the sensitivity of the Hemoccult test is relatively low and that the relatively short sojourn time is in favour of annual screening. © 2001 Cancer Research Campaign http://www.bjcancer.com

**Keywords**: colorectal neoplasm; mass-screening; sensitivity; sojourn time; statistical models

Colorectal cancer meets requirements justifying mass screening (European Group for Colorectal Cancer Screening, 1999). Colorectal cancer is usually preceded for many years by an asymptomatic adenoma; the endoscopic resection of adenomas decreases colorectal cancer incidence between 85% and 90% (Winawer et al, 1993). Currently, the faecal occult blood test HemoccultII® is the only efficient test for screening. 3 prospective controlled cohort studies in well-defined European populations aged 45 to 74 years have demonstrated a decrease in specific colorectal cancer mortality between 14% and 18% with a biennial HemoccultII® test and a median follow-up of 8 to 10 years (Hardcastle et al, 1996; Kronborg et al, 1996; Faivre et al, 1999).

Besides determining the incidence of interval cancers by age-sex-subsite and time since a negative screen, this study aimed to estimate HemoccultII® test sensitivity and mean sojourn time from a screening programme based on a biennial test (6 campaigns) in a well-defined French population covered by the Burgundy registry of digestive tract cancers (Faivre et al, 1999).

### MATERIALS AND METHODS

The study design has been described previously (Tazi et al, 1997). All residents ($n$ = 45 603) of 12 administrative districts of the department of Saône-et-Loire (Burgundy, France), born between 1914 and 1943 (aged 45–74 years), were invited to participate in a mass screening programme for colorectal cancer. A faecal occult blood test, the HemoccultII® test (SKD, France), was used as a screening test. The first round of mass screening took place in 1988 or 1989. The screening rounds were repeated for the whole population in 1990, 1992, 1994, 1996 and 1998. All data were recorded from 1 January 1988 until 31 December 1998, the closing date of the study. A colonoscopy was offered if the test was positive.

Subsite was defined for each cancer as: right colon (from the caecum to the transverse colon), left colon (from the splenic flexure to the sigmoid) or rectum (from the recto-sigmoidal junction to the rectal ampulla). In the screening programme population, cancers were classified in 3 groups: cancers diagnosed from a positive HemoccultII® test, interval cancers diagnosed after a negative HemoccultII® test and cancers in those who did not participate in any of the 6 screening rounds. Adenomas detected by screening were classified according to the site (as for cancers in 3 groups) and size (millimetres). Polyps other than adenomas were excluded.

### Statistical methods

The statistical approach used in this article followed the same line of thinking as that in previous publications treating the same problem for breast cancer screening and mammography (Day and Walter, 1984; Day 1985; Paci and Duffy, 1991). For the prevalent screen (first screen attended by a participant), the cancer prevalence at screening was compared to the corresponding age-sex-subsite-specific control population incidence rate, through the prevalence/incidence ratio. This ratio gave a rough estimate of the product of the mean sojourn time by the sensitivity (Day, 1985). A second approach evaluated the incidence of interval cancer as a function of time since the last negative screen and this incidence was compared to the expected incidence in the absence of screening (i.e. control population incidence). More precisely, all participants with a negative test at either a prevalent or an incident

*Received 18 September 2000*
*Revised 14 February 2001*
*Accepted 16 February 2001*

*Correspondence to:* J Faivre

**Table 1** Incidence[a] of colorectal cancer in the department of Saône-et-Loire without screening for people aged 45–75 years and more, according to subsite, sex and age

| Age (years) | Right colon | Left colon | Rectum | Subsite unknown | Overall |
|---|---|---|---|---|---|
| Males | | | | | |
| 45–49 | 4.3 | 6.4 | 12.8 | 0.0 | 23.4 |
| 50–54 | 9.9 | 16.8 | 31.7 | 0.0 | 58.5 |
| 55–59 | 15.8 | 31.6 | 53.3 | 4.0 | 104.7 |
| 60–64 | 23.6 | 50.5 | 88.7 | 4.5 | 167.4 |
| 65–69 | 65.6 | 85.1 | 102.8 | 3.5 | 257.0 |
| 70–74 | 62.5 | 99.3 | 184.2 | 1.6 | 347.5 |
| ≥ 75 | 91.3 | 167.2 | 221.1 | 7.7 | 489.5 |
| Females | | | | | |
| 45–49 | 3.2 | 8.7 | 10.8 | 1.1 | 23.8 |
| 50–54 | 2.9 | 21.5 | 14.7 | 1.0 | 40.1 |
| 55–59 | 14.1 | 30.2 | 23.6 | 0.9 | 68.8 |
| 60–64 | 17.4 | 36.9 | 42.1 | 1.0 | 97.5 |
| 65–69 | 37.9 | 58.3 | 40.8 | 0.0 | 137.0 |
| 70–74 | 57.0 | 57.0 | 55.8 | 0.0 | 169.8 |
| ≥75 | 94.9 | 77.1 | 80.0 | 1.8 | 254.9 |

[a]Incidence rate per 100 000 using 1982–1987 Digestive Cancer Registry data.

screen were considered at risk of cancer until the next screen, death or occurrence of a colo-rectal cancer. Person-years at risk broken down by age, sex, type of screen, and time since screen were calculated using the STATA statistical software and its survival procedures (StataCorp. 1999. Stata Statistical Software: Release 6.0. College Station, TX: Stata Corporation). Expected incidence was then calculated by applying the age-sex-subsite-specific rate of the control population (Table 1). The above-cited references show that the ratio observed/expected cancers after a negative test provides information on test sensitivity and the distribution of sojourn time. This ratio is known as proportional incidence. In particular, this ratio calculated in a short period after a negative screen test is a rough approximation of one minus the sensitivity: the higher the ratio the lower the sensitivity (Moss et al, 1999). As shown in the appendix, the information on the sojourn time is mainly contained in the increase of this ratio with time since screening. In order to work with independent observations, we used the formula:

$$Log\left(1-\frac{O_{t,\,\Delta t}}{E_{t,\,\Delta t}}\right) = Log\,(S) - \lambda t$$

where $O_{t,\Delta t}$ and $E_{t,\Delta t}$, are respectively observed and expected cancer within the intervals $]t - \Delta t;\ t + \Delta t[$, and S is the sensitivity of the test and $\lambda$ the inverse of the mean sojourn time (see Appendix). We then obtained estimates of $S$ and $\lambda$ through a simple weighted least-square regression based on observed data in short intervals following a negative screen. In principle we should have taken into account the screen performed before the last negative screen and entered into our formula the fact that a cancer could have been missed already by the previous screening. This refinement is of interest but would have had little effect on our results given that only a few cancers have a sojourn time larger than 2 years and that few persons in the cohort attended all screening rounds.

### RESULTS

The number of screened individuals and the number of detected colorectal cancers by sex at each screening round are detailed in Table 2. From the 45 603 individuals of the study cohort only 11 851 (26.0%) attended all screening rounds, but 31 664 individuals (69.4%) attended at least one round. Compliance at each round was higher for women. At the end of the study 195 colorectal cancers were detected, 128 in men (65.6%) and 67 in women (34.4%). Interval cancers were diagnosed in 294 individuals, among whom 6 were diagnosed after a negative test in the last round for which the follow-up was shorter. There were 171 interval cancers in men (58.2%) and 123 in women (41.8%). In men 125 interval cancers (73.1%) were diagnosed within 2 years of a negative screen. In women there were 95 such interval cancers (77.2%).

Table 3 shows the cancers detected at prevalent screens by sex and subsite. From this table we can see that the ratio observed/ expected was significantly higher in men than in women ($\chi^2$ = 5.44; $P$ = 0.02). In men there was some evidence of heterogeneity between subsites suggesting that the sojourn time was longer or that sensitivity was higher, or both, for the left colon than for the other subsites ($\chi^2$ = 3.85; $P$ = 0.05 if the left colon is compared to the other sites). There is no such difference in women but the number of cases is too small to interpret this result further.

Table 4 analysed the incidence of the interval cancers by subsite: the proportional incidence was lower for the left colon than for the other subsites and increased between year 1 and year 2 confirming both the above result from prevalence analysis and what is known from the literature. However the differences were not significant. There was no difference in proportional incidence between sexes (0.61 for males and 0.64 for females). Although some differences were seen for age (0.67 for peoples aged 45–64 years and 0.59 for those aged under 65 years) and type of screen (0.70 for first screen and 0.59 for rescreen), none of them were significant.

The joint estimates of $S$ and $\lambda$ were obtained from the results shown in Table 5. We performed a regression as explained in the method section (equation 3) on the first 3 values of $O_t/E_t$ for which an increase of the proportional incidence is seen. This approach gave an estimate of $S$ equal to 0.57 (SE = 0.10) and an estimate of $\lambda$ equal to 0.39 (SE = 0.19) corresponding to a mean sojourn time of 2.56 years. When different parameters for men and women were

 *© 2001 Cancer Research Campaign*

**Table 2** Screened population and screened detected colorectal cancers by screening round and sex

| Screening round | Number screened | | | Cancers detected | | |
|---|---|---|---|---|---|---|
| | Male | Female | Both sexes | Male | Female | Total |
| 1 | 10 770 | 13 288 | 24 058 | 30 | 10 | 40 |
| 2 | 10 590 | 13 070 | 23 660 | 13 | 11 | 24 |
| 3 | 10 512 | 13 172 | 23 684 | 25 | 13 | 38 |
| 4 | 9 975 | 12 659 | 22 634 | 22 | 12 | 34 |
| 5 | 9 204 | 11 823 | 21 027 | 22 | 11 | 33 |
| 6 | 8 177 | 10 551 | 18 728 | 16 | 10 | 26 |
| Total | 59 228 | 74 563 | 133 791 | 128 | 67 | 195 |

**Table 3** Observed and expected[a] number of colorectal cancers after the first positive test (prevalent screening) by sex and subsite

| Sex | Subsite | Observed screened cases (O) | Expected cases (E) | Ratio O/E |
|---|---|---|---|---|
| Males | | | | |
| | Right colon | 9 | 4.5 | 2.0 |
| | Left colon | 23 | 8.0 | 2.9 |
| | Rectum | 19 | 12.3 | 1.5 |
| | All sites | 51 | 24.8 | 2.1 |
| Females | | | | |
| | Right colon | 6 | 4.9 | 1.2 |
| | Left colon | 8 | 6.6 | 1.2 |
| | Rectum | 6 | 6.2 | 1.0 |
| | All sites | 20 | 17.7 | 1.1 |
| Both sexes | | | | |
| | Right colon | 15 | 9.4 | 1.6 |
| | Left colon | 31 | 14.5 | 2.1 |
| | Rectum | 25 | 18.6 | 1.3 |
| | All sites | 71 | 42.6 | 1.7 |

[a]Age-standardized expected number of cases without screening using 1982–1987 Saône-et-Loire Digestive Cancers Registry data.

**Table 4** Proportional incidence of colorectal cancer after a negative test by subsite

| Subsite | Person-years | Observed interval cases (O) | Expected cases (E) | Proportional incidence O/E |
|---|---|---|---|---|
| Rectum | | | | |
| Year 1 | 126 313 | 52 | 82.8 | 0.63 |
| Year 2 | 99 868 | 46 | 68.0 | 0.68 |
| Total | 226 181 | 98 | 150.8 | 0.65 |
| Left colon | | | | |
| Year 1 | 126 313 | 29 | 66.7 | 0.43 |
| Year 2 | 99 868 | 38 | 54.6 | 0.70 |
| Total | 226 181 | 67 | 121.3 | 0.55 |
| Right colon | | | | |
| Year 1 | 126 313 | 33 | 44.4 | 0.74 |
| Year 2 | 99 868 | 21 | 36.7 | 0.57 |
| Total | 226 181 | 54 | 81.1 | 0.67 |

Subsite is unknown for 1 interval cancer.

estimated we obtained S equal to 0.57 and MST equal to 3.21 for men and 0.63 and 1.51 respectively for women. In both cases the negative correlation of these 2 parameters was large and the individual estimate not very reliable. In contrast they provided a reasonable estimate of the product S.MST. This latter parameter, 1.83 in men and 0.95 in women, is in broad agreement with the estimates obtained from the prevalence/incidence ratio.

The sensitivity of the screening programme was calculated for 1 and 2 years time intervals following a negative test. The overall sensitivity of the screening programme was 0.61 within 1 year and 0.43 within 2 years (169 screened-detected and 220 interval cancers). The sensitivity of the screening programme according to screening round within 1 year after a negative test is given in Table 6. It was slightly higher after the first screening round than after the following ones.

### DISCUSSION

Screening for colorectal cancer with the HemoccultII® test has proved to be efficacious through 2 population-based intervention trials (Hardcastle et al, 1996; Kronborg et al, 1996) and one intervention based on a selected group of volunteers (Mandel et al, 1993). A recent meta-analysis estimates that the reduction in mortality may be in the range of 16% to 20% (Towler et al, 2000) a relatively small benefit. In the 2 above-cited population-based trials the sensitivity of the programme among the participants was 55% in Funen (Gyrd-Hansen et al, 1997) and 51% in Nottingham (Moss et al, 1999). The data of the present intervention led to results of the same order of magnitude but slightly smaller (43.4%). In contrast with this broad agreement on efficacy there is a wide range in the various sensitivity estimates of the test (Gyrd-Hansen et al, 1997; Launoy et al, 1997; Moss et al, 1999) ranging from 22% to 90% (Moss et al, 1999). Even if we restrict the review to population-based study we obtain a large range of estimates (34% to 75%). One obvious reason for these discrepancies lies in the lack of a uniform definition of sensitivity. If sojourn time starts when the cancer bleeds, the sensitivity of the test is the probability that the cancer is bleeding at the time of the test. It is unlikely that this quantity is constant over the sojourn time, thereby bringing into question the adequacy of the model. If we accept its use, the resulting estimate of sensitivity should be considered as the portion of the sojourn time during which the cancer is bleeding. With this caveat in mind our findings can be compared with results obtained elsewhere. All studies agree in showing that screening is more efficient for detecting tumours in the left colon. In practice it is more easy to diagnose distal than proximal tumours and colonoscopy may fail to explore the entire colon. In the present study, the colonoscopy was not performed after a positive test in 412 cases (20.7%) and did not go beyond the hepatic flexure in 134 cases (6.8%). As a consequence the sensitivity of the procedure is more limited for the right colon than for other sites. The observed difference in proportional incidence and in the prevalence/

© 2001 Cancer Research Campaign 

**Table 5** Proportional incidence of colorectal cancer after a negative test

| | Time since a negative screen (years) | | | | | |
|---|---|---|---|---|---|---|
| | **0.5** | **1** | **1.5** | **2** | **2.5** | **3** |
| Males | | | | | | |
| Observed interval cases | 29 | 36 | 30 | 30 | 11 | 3 |
| Expected cases | 59.3 | 55.2 | 49.2 | 45.0 | 12.9 | 6.9 |
| Σ Obs/ Σ Exp | 0.49 | 0.57 | 0.58 | 0.60 | 0.61 | 0.61 |
| Females | | | | | | |
| Observed interval cases | 24 | 25 | 28 | 18 | 6 | 3 |
| Expected cases | 42.4 | 39.4 | 35.0 | 32.0 | 9.7 | 5.1 |
| Σ Obs/ Σ Exp | 0.57 | 0.60 | 0.66 | 0.64 | 0.64 | 0.64 |
| Both sexes | | | | | | |
| Observed interval cases | 53 | 61 | 58 | 48 | 17 | 6 |
| Expected cases | 101.7 | 94.6 | 84.2 | 77.0 | 22.6 | 12.0 |
| Σ Obs/ Σ Exp | 0.52 | 0.58 | 0.61 | 0.62 | 0.62 | 0.62 |

Σ Obs: cumulated observed interval cases; Σ Exp: cumulated expected cases.

**Table 6** Estimate of test sensitivity by screening round according to interval cancers occurring within one year after a negative test

| Screening round | Screen-detected cancers | Interval cancers within one year | Sensitivity (%) |
|---|---|---|---|
| 1 | 40 | 15 | 72.7 |
| 2 | 24 | 23 | 51.1 |
| 3 | 38 | 30 | 55.9 |
| 4 | 34 | 15 | 69.4 |
| 5 | 33 | 25 | 56.9 |
| Total | 169 | 108 | 61.0 |

incidence ratio may therefore be explained by a lower sensitivity of the test rather than by a shorter sojourn time. In contrast the studies disagree on the size of the difference between male and female and in the direction of this difference. Proportional incidence is slightly higher in females and increases with time since screening in both the Funen study (Gyrd-Hansen et al, 1997) and our study. It is significantly smaller in females than in males in the Nottingham study and does not increase with time. The Calvados results (Launoy et al, 1997) are qualitatively similar to those of Nottingham. It is difficult to understand these discrepancies but the random fluctuations of the number of cases are too large to permit a more satisfactory analysis.

There are few reports on the analysis of the prevalence/incidence ratio for colo-rectal cancer screening. In the Funen programme, the ratio was close to one and slightly higher for men than for women. In the above Calvados study, the prevalence/incidence ratio was calculated differently and does not directly provide an S.MST estimate, but this can be inferred as smaller in value and slightly higher for males. In our study the ratio is also greater for men and the results are in broad agreement with those obtained from the proportional incidence analysis.

When using the prevalence/incidence ratio to estimate S.MST we were not able to take into account prevalence or incidence of adenoma. Therefore an estimate refers only to the MST of the tumour when it has become malignant and to the sensitivity of the test to detect cancer. On the other hand the cumulative incidence of interval cancer is influenced by the ability of the test to detect adenoma. However the duration of the adenoma-cancer sequence is considered to be longer than 10 years. Therefore the part of the cumulative incidence which is used in our calculation is only influenced by the ability of the test to detect cancer. As a consequence, both approaches for estimation of S and MST refer to the asymptomatic cancer part of the sojourn time. Although it is necessary to assess the efficacy of Hemoccult to decrease the incidence of cancer through the detection of adenoma, our approaches and data did not permit this evaluation.

The sensitivity of the programme among participants is lower in Burgundy than in either Denmark or England. Among other explanations, differences in the sensitivity of the test and in sojourn time may be relevant. In particular the increase in sojourn time may be due to delayed diagnosis of symptomatic cancer, which in turn could explain a larger benefit of screening. It is therefore important to have information on these 2 parameters. Unfortunately all studies up to now have demonstrated the difficulties in obtaining reliable estimates of sensitivity and mean sojourn time due to their strong inverse relation and the relatively small study sizes. One other reason for the lower sensitivity of the programme in Burgundy is the relatively weak compliance for only 26% of the cohort attended all the screening rounds. This explanation is consistent with the relatively high proportion of Hemoccult detected cancers among the interval cancer in the first year after a negative screen (61%).

Several simple estimates of sensitivity have been proposed, including the proportion among the total of those detected on a positive screen or diagnosed on symptoms within one year of a negative screen. These estimates are given in Table 6 and are not too far from those obtained with the simple regression performed on proportional incidence. We think on the contrary that the

*© 2001 Cancer Research Campaign*

estimate based on $1–O_t/E_t$ is generally too low especially if the sojourn time is exponentially distributed: with a 2-year mean sojourn time, 40% of cancers have a sojourn time less than 1 year.

For the practical purpose of managing and designing mass screening programmes we consider that the sensitivity of the Hemoccult test is near to 60% and that the mean sojourn time is about 2 years, but these estimates need to be refined in more precise studies.

### APPENDIX

If we were to suppose that in a given age-sex-subsite category the incidence rate in the absence of screening is constant, the probability that a new cancer surfaces within the interval ]0,t] after a negative screen is:

$$(1-e^{-lt}) \times \int_0^t \frac{1}{t} Pr(ST \leq u)du \qquad (1)$$

I is the constant incidence of cancer in the absence of screening and ST is the sojourn time. The integral is the sum of the probability of occurrence of a cancer with a sojourn time less than *u* at time *u*; the first factor is the probability of occurrence of a cancer of the given age-sex-subsite within the interval ]0,t].

All other cancers with a sojourn time greater than *u* could have been detected or missed with a probability *S* and *1–S* respectively. Therefore the probability of observing an incident cancer in the interval ]0,t] is:

$$CI\,(t) = \frac{(1-e^{-1t})}{t} \times \left[(1-S)\int_0^t (1-F(u))du + \int_0^t F(u)du\right]$$
$$= \frac{(1-e^{-1t})}{t} \times \left[(1-S)t + S\int_0^t F(u)du\right]$$

where $F(u)$ is Pr $(ST \leq u)$.

When $I \times t$ is small, the first factor is well approximated by *I* itself. Moreover if we believe that an exponential distribution for the sojourn time is acceptable the formula simplifies and we obtain the cumulative distribution of interval cancer after a negative screen as:

$$CI\,(t) = I \times \left[t + \frac{S}{\lambda}(e^{-\lambda t} - 1)\right] \qquad (2)$$

where $\lambda$ is the inverse of the mean sojourn time.

From this formulation we can see that the ratio of the interval cancer incidence to that expected in the absence of screening, known as the proportional incidence, is given by:

$$O_t / E_t = 1 + S\,\frac{e^{-\lambda t} - 1}{\lambda t}$$

which for small $\lambda t$ is approximated by $1–S + S\lambda\, t/2–S\lambda^2\, t^2/6$. It is possible to estimate this distribution function from its density by taking the derivative of (2): The number observed in a small interval around *t* is proportional to $I\Delta t \times (1–Se^{-\lambda t}) = E_{\Delta t} \times (1–Se^{-\lambda t})$. Therefore:

$$Log\left(1 - \frac{O_{t,\,\Delta t}}{E_{t,\,\Delta t}}\right) = Log\,(S) - \lambda t \qquad (3)$$

### ACKNOWLEDGEMENTS

This project was funded by the Europe Against Cancer Programme, INSERM, the Fond National de Prévention, the Burgundy Regional Council and the French League Against Cancer.

Dr JL Jouve was supported by the Fondation de France.

### REFERENCES

Day NE (1985) Estimating the sensitivity of a screening test. *J Epidemiol Commun Health* **39**: 364–366

Day NE and Walter SD (1984) Simplified models of screening for chronic disease from mass screening programmes. *Biometrics* **40**: 1–14

European Group for Colorectal Cancer Screening (1999) Recommandation to include colorectal cancer screening in public health policy. *J Med Screen* **6**: 80–81

Faivre J, Tazi MA, Milan C, Lejeune C, Dassonville F and Durand G (1999) L'étude bourguignonne d'évaluation du dépistage de masse du cancer colorectal par la recherche d'un saignement occulte dans les selles: résultats à 9 ans. *Gastroenterol Clin Biol* **23**: A88

Gyrd-Hansen D, Søgaard J and Kronborg O (1997) Analysis of screening data: colorectal cancer. *Int J Epidemiol* **26**: 1172–1181

Hardcastle JD, Chamberlain JO, Robinson MHE, Moss SM, Amar SA, Balfour TW, James PD and Mangham CM (1996) Randomized controlled trial of faecal-occult-blood screening for colorectal cancer. *Lancet* **348**: 1472–1477

Kronborg O, Fenger C, Olsen J, Dan Jørgensen O and Søndergaard O (1996) Randomized study as screening for colorectal cancer with faecal-occult-blood test. *Lancet* **348**: 1467–1471

Launoy G, Smith TC, Duffy SW and Bouvier V (1997) Colorectal cancer mass-screening: estimation of faecal occult blood test sensitivity, taking into account cancer mean sojourn time. *Int J Cancer* **73**: 220–224

Mandel JS, Bond JH, Church TR, Snover DC, Bradley GM, Schuman LM and Ederer F (1993) Reducing mortality from colorectal cancer by screening for fecal occult blood. Minnesota Colon Cancer Control Study. *N Engl J Med* **328**: 1365–1371

Moss SM, Hardcastle JD, Coleman DA, Robinson MHE and Rodrigues VC (1999) Interval cancers in a randomized controlled trial of screening for colorectal cancer using a faecal occult blood test. *Int J Epidemiol* **28**: 386–390

Paci E and Duffy SW (1991) Modelling the analysis of breast cancer screening programme: Sensitivity, Lead Time and Predictive Value in the Florence District Programme (1975–1986). *Int J Epidemiol* **20**: 4852–4858

Tazi MA, Faivre J, Dassonville F, Lamour J, Milan C and Durand G (1997) Participation in faecal occult blood screening for colorectal cancer in a well defined French population: results of five screening rounds from 1988 to 1996. *J Med Screen* **4**: 147–151

Towler BP, Irwig L, Glasziou P, Weller D and Kewenter J (2000) Screening for colorectal cancer using the faecal occult blood test, Hemoccult. *Cochrane Database Syst Rev* **2**: CD001216

Winawer SJ, Zauber AG, Ho MN, O'Brien MJ, Gottlieb LS, Sternberg SS, Waye JD, Schapiro M, Bond JH and Panish JF (1993) The National Polyp Study Workgroup. Prevention of colorectal cancer by colonoscopic polypectomy. *N Engl J Med* **329**: 1977–1981

*© 2001 Cancer Research Campaign*
